# MDSC suppresses T cell antitumor immunity in CAC via GPNMB in a MyD88‐dependent manner

**DOI:** 10.1002/cam4.6887

**Published:** 2023-12-22

**Authors:** Bo Wang, Lu Wang, Runshi Shang, Lin Xie

**Affiliations:** ^1^ Department of Gastroenterology, Tongji Hospital, Tongji Medical College Huazhong University of Science and Technology Wuhan China; ^2^ Institute of Organ Transplantation, Tongji Hospital, Tongji Medical College, Huazhong University of Science and Technology; Key Laboratory of Organ Transplantation, Ministry of Education; NHC Key Laboratory of Organ Transplantation; Key Laboratory of Organ Transplantation, Chinese Academy of Medical Sciences Wuhan China

**Keywords:** colitis‐associated colorectal cancer, GPNMB, MDSC, MyD88 signaling, T cell immunity

## Abstract

**Background:**

Myeloid‐derived suppressor cells (MDSCs) played an essential role in tumor microenvironment to suppress host antitumor immunity and help cancer cells escape immune surveillance. However, the molecular mechanism behind tumor evasion mediated by MDSCs is not fully understood. Glycoprotein nonmetastatic melanoma protein B (GPNMB) is considered to associate with tumor initiation, metastasis and angiogenesis. Blocking GPNMB function is a potentially valuable therapy for cancer by eliminating GPNMB^+^MDSCs. Our previous study has proved that blockage the MyD88 signaling with the MyD88 inhibitor, TJ‐M2010‐5, may completely prevent the development of CAC in mice, accompanying with downregulation of GPNMB mRNA in the inhibitor‐treated mice of CAC.

**Methods:**

We here focus on the underlying the relationship between GPNMB function and MyD88 signaling pathway activation in MDSCs' antitumor activity in CAC.

**Results:**

CAC development in the mouse model is associated with expanded GPNMB^+^MDSCs by a MyD88‐dependent pathway. The GPNMB expression on MDSCs is associated with MyD88 signaling activation. The inhibitory effect of MDSCs on T cell proliferation, activation and antitumor cytotoxicity in CAC is mediated by GPNMB in a MyD8‐dependent manner.

**Conclusion:**

MyD88 signaling pathway plays an essential role in GPNMB^+^MDSC‐mediated tumor immune escape during CAC development and is a promising focus for revealing the mechanisms of MDSC that facilitate immunosuppression and tumor progression.

## 1 INTRODUCTION

Colorectal cancer remains the leading incidence and mortality rates around the world for decades. Inflammatory bowel disease is a crucial trigger for the occurrence of colorectal cancer.^1^ Chronic inflammation of the colon causes intestinal cells damages and genetic mutations, and activates the intestinal immune system. The interaction between mutated cancer cells and host immune system in the colon, where is called tumor microenvironment (TME), promotes the development of colorectal cancer. Multiple molecules, mediators, and cells participate in the construction of TME, which contributes to immune escape, expansion, and metastasis of tumor cells.^2^

Myeloid‐derived suppressor cells (MDSCs) have been confirmed to be one of the key players engaged in TME to inhibit host antitumor immunity and accelerate tumor progression. Many studies have declared that MDSCs are one of potential therapy for cancer treatment. Currently, multiple treatment approaches targeting MDSCs have been examined in preclinical and clinical studies.^3^ In colitis‐associated colorectal cancer (CAC), MDSCs massively expand and infiltrate the TME.^4^ Decreasing the expanding MDSCs or attenuating their immunosuppressive function in host is an important therapeutic method to prevent the development of CAC.

The Toll‐like receptor (TLR) pathway plays an important role during inflammation and cancer development and its activation through sensing of danger‐associated molecular patterns (DAMP) released in TME helps host initiate anti‐inflammatory and antitumor immune response.^5^ Myeloid differentiation factor 88 (MyD88) is a central adaptor‐signaling molecule for most TLRs except TLR3. Studies have indicated that expansion and suppressive activity of MDSC depend on TLR signaling through the adaptor molecule MyD88.^6^, ^7^, ^8^, ^9^ Blocking Myd88 signaling has been proved to induce antitumor effects by reversing the immunosuppressive effect of MDSCs.^10^, ^11^

Glycoprotein nonmetastatic melanoma protein B (GPNMB) is a glycosylated type I transmembrane protein. It is expressed in a variety of cell types, such as melanoma, glioblastoma, dendritic cells (DCs), macrophages (M_Φ_), and MDSCs in the immune system.^12^, ^13^ There is growing evidence supporting that GPNMB is overexpressed among various cancers and it exhibits immunosuppressive function to promote tumor growth and metastasis.^14^, ^15^ GPNMB is considered to be an applicable marker to identify MDSCs with suppressive activity^16^ and blocking GPNMB function is a potentially valuable therapy for colorectal cancer by eliminating GPNMB^+^ MDSCs.^17^ Our previous study has proved that the administration of TJ‐M2010‐5 (a patented MyD88 inhibitor, synthesized by Dr. Zhou's group) may completely prevent the development of CAC in mice,^18^ accompanying with downregulation of GPNMB mRNA in the inhibitor‐treated mice of CAC. We here focus on the underlying the relationship between GPNMB function and MyD88 signaling pathway activation in MDSCs' antitumor activity in CAC.

## 2 METHODS

### 2.1 Animals

Six‐week old wild type (WT) and MyD88^−/−^ BALB/c female mice, SPF grade, were purchased from Gempharmatech Company (Nanjing, China). The experimental operation has been approved by the Animal Care and Research Committee of Huazhong University of Science and Technology.

### 2.2 CAC model

The mouse model of CAC was induced as previously described.^18^ Briefly, each mouse was injected intraperitoneally (*i.p*.) with 10 mg/kg azoxymethane (AOM, Sigma‐Aldrich Chemical, Germany). One week later, the mice were fed with 3 cycles of 2.5% dextran sodium sulfate (DSS, Meilunbio, China) solution for 1 week and 2 weeks of plain drinking water throughout a 10‐week observation period. A group of sex‐ and age‐matched normal control (NC) mice received plain drinking water. Samples [spleen, peripheral blood (PB), and bone marrow (BM)] were collected at 10 weeks (W10).

### 2.3 MyD88 inhibitor (TJ‐M2010‐5) treatment

All WT mice were randomly divided into three groups: NC, CAC model (CAC), and MyD88 inhibitor‐treated CAC (Inhibitor‐CAC) groups. TJ‐M2010‐5 was *i.p*. administered to mice in the Inhibitor‐CAC group daily at 50 mg/kg body weight for 10 weeks beginning 2 days before the first DSS administration.

### 2.4 Preparation of Splenocytes, bone marrow cells (BMCs), and peripheral blood cells (PBCs) suspensions

Splenocytes: spleens of mice were removed, diced in phosphate buffer saline (PBS) and pressed through a 70 μM strainer, and the harvested cells were washed with PBS and suspended. BMCs: femurs and tibia of mice were aseptically cut and debrided of surrounding skeletal muscle and other tissue, and BMCs were flushed from the femur and tibia using PBS and strained through a 100 μM strainer. PBCs: Blood was drawn from the inner canthus of mice. PBCs were suspended after the red blood cells were removed from single‐cell suspensions with red blood cells lysis solution (Solarbio Comp., Beijing, China).

### 2.5 Flow cytometry analysis

The following antibodies (Abs) were used for flow cytometry analysis: CD11b and CD4 from BD Biosciences (USA); Gr‐1 from Biolegend Inc (USA); GPNMB, CD8a, and CD107a from ThermoFisher Scientific (USA). The cells were surface‐labeled with anti‐mouse Abs for 30 min at 4°C in the dark, and subsequently washed, fixed, and permeabilized by the Cytofix/Cytoperm Solution Kit (BD Biosciences, USA). The permeabilized cells were labeled by anti‐mouse CD107a for 30 min at 4°C in the dark. The cells were read using a FACS Celesta flow cytometer, and data were analyzed by FlowJo software (Version 10.0).

### 2.6 Quantitative real‐time polymerase chain reaction (RT‐qPCR)

Total RNA was isolated from cells using TRIzol reagent (Invitrogen, USA). 2 μg of RNA were used for cDNA synthesis with a Hifair II 1st Strand cDNA Synthesis kit (Yeasen Biotech., China). cDNA was amplified with the Hieff qPCR SYBR Green Master Mix (Yeasen Biotech., China). The sequences of the primers are followed: GPNMB forward primer (5’‐AATGGGTCTGGCACCTACTG‐3′, reverse primer (5’‐GGCTTGTACGCCTTGTGTTT‐3′); β‐actin forward primer (5′‐AATCGTGCGTGACATCAAAGA‐3′, reverse primer (5′‐CCATACCCAAGAAGGAAGGC‐3′). qRT‐PCR was performed on a StepOne System (Life Technologies, USA) according to the manufacturer's instructions. The relative mRNA expression levels were determined using the ΔΔCT calculation method. The mRNA expression in each sample was normalized to the internal control β‐actin.

### 2.7 Cell sorting

CD11b^+^Gr‐1^+^ MDSCs, CD11b^−^ BMCs, CD4^+^, and CD8^+^ splenic T cells were isolated by magnetic beads and columns according to the manufacturers' instructions (MiltenyiBiotec., Germany).

### 2.8 MDSC suppression assay on T cells proliferation and activation

CD11b^+^Gr‐1^+^ MDSCs sorted from BMCs of mice and 5 × 10^5^ CD4^+^ or CD8^+^ T cells sorted from autologous splenocytes were cocultured in 48‐well plates in complete RPMI medium at ratios of 1:1. T cells activation was performed by means of anti‐CD3/anti‐CD28‐coated microbeads (ThermoFisher Scientific, USA) activation for 4 days. The T cell proliferation was performed using carboxy‐fluorescein diacetate succinimidyl ester (CFSE, ThermoFisher Scientific, USA) staining assay. Suppressor ability of MDSCs was indicated as percentage of suppression: 1 – *b/a* × 100%, where *a* is the proliferation rate of T cells only; and *b* is the proliferation rate of T cells in cocultures at 1:1 ratio. IFN‐γ levels in the cultures were assessed using ELISA Kit (BOSTER Biological Technology Co., China) for activation assay.

### 2.9 Antitumor cytotoxicity of CD8 ^+^ T cells

Sorted splenic CD8^+^ T cells were cocultured with target tumor cells (CT26.WT, BOSTER Biological Technology Co., China, 3rd to 10th passage) labeled with CFSE (Biolegend Inc, USA) at 5:1 ratio w/o sorted CD11b^+^Gr‐1^+^ MDSCs from BMCs of mice with CAC, in RPMI 1640 complete medium (Gibco, USA). After 72 h, CD8^+^ T cell cytotoxicity was measured by the expression of effector molecules, such as CD107a, on activated CD8^+^ T cells and mortality of target tumor cells by Zombie Aqua (Biolegend Inc, USA) staining by FACS.

### 2.10 MDSC differentiation from BM‐derived immature myeloid cells in vitro

CD11b^−^ cells sorted from BMCs were cultured in the presence of 10 ng/mL of GM‐CSF (Peprotech, USA) and 1 μg/mL LPS (Sigma‐Aldrich Chemical, Germany) in RPMI 1640 complete medium supplemented with sodium‐pyruvate (1 mM, Meilunbio, China) and β‐mercaptoethanol (50 μM, Solarbio, China) w/o 20 μM MyD88 inhibitor administration. At 8 days of cultivation, the population of CD11b^+^Gr‐1^+^ MDSC was calculated to express the differentiation of MDSC from immature myeloid cells. GPNMB expression on MDSC were detected by flow cytometry, and the mRNA expression of GPNMB on induced MDSCs were detected by RT‐qPCR.

### 2.11 Statistical analyses

Comparisons of means ± standard deviation (SD) between groups were performed using Student's *t*‐test and one‐way analysis of variance with GraphPad Prism softwar (version 9.0; La Jolla, USA). *p‐*value <0.05 were considered statistically significance.

## 3 RESULTS

### 3.1 GPNMB‐expressing MDSCs are expanded in the mouse model of CAC by a MyD88‐dependent pathway

**FIGURE 1:**
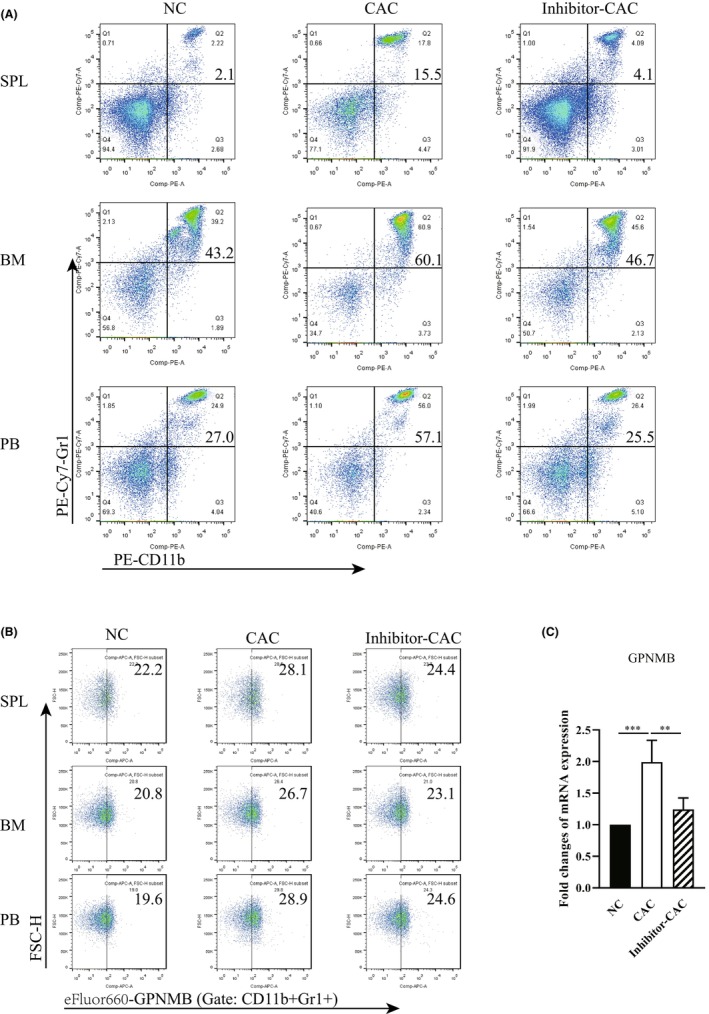
GPNMB^+^ MDSCs are expanded in the mouse model of CAC by a MyD88‐dependent pathway. (A) Population of CD11b^+^Gr‐1^+^ MDSCs in the spleen (SPL), BM, and PB by flow cytometry 10 weeks after induction in the normal control (NC), the CAC group (CAC) and the MyD88 inhibitor‐treated CAC (Inhibitor‐CAC) groups. (B) Population of GPNMB^+^ MDSCs (gated on CD11b^+^Gr‐1^+^ cells) in the SPL, BM, and PB by flow cytometry 10 weeks after induction in the NC, CAC, and Inhibitor‐CAC groups. (C) Relative levels of GPNMB mRNA transcripts in BMCs from mice with CAC examined by RT‐qPCR. Data of RT‐qPCR were normalized to β‐Actin expression in each sample, and the relative expression levels were compared with that in NC group. **p < 0.01; ***p < 0.001. *N* = 5 per group. Data are expressed as the mean ± SD of each group.

Several reports have shown that patients suffering types of cancer display highly expanded GPNMB^+^ MDSCs in the blood, such as melanoma, lung, colorectal, kidney, pancreatic, breast, bladder, or prostate cancer.^14^ Here, we detected the molecular mechanism of induction of GPNMB^+^ MDSCs expansion in the AOM/DSS‐induced CAC mice model. Splenocytes, BMCs, and PBCs from mice with CAC were analyzed by flow cytometry for CD11b and Gr‐1 expression. CD11b^+^Gr‐1^+^ MDSCs were sorted for GPNMB expression. There was significant expansion of CD11b^+^Gr‐1^+^ MDSCs (Figure 1A) in the spleen (15.5 ± 4.2%, *p* = 0.0000), the BM (60.1 ± 4.5%, *p* = 0.0000) and the PB (57.0 ± 15.6%, *p* = 0.0000) of mice with CAC, compared with normal control (NC) mice (2.1 ± 0.5%, 43.2 ± 4.7%, and 27.0 ± 11.2% respectively). For GPNMB^+^ cells among MDSCs, mice with CAC exhibited higher GPNMB‐positivity (28.1 ± 2.8% in the spleen, 26.7 ± 1.3% in the BM, and 28.9 ± 1.4% in the blood), in contrast to 22.2 ± 1.6% (spleen, *p* = 0.0000), 20.8 ± 1.6% (BM, *p* = 0.0000), and 19.6 ± 2.9% (blood, *p* = 0.0000) in NCs (Figure 1B). While after treatment with the MyD88 inhibitor to block MyD88 signaling pathway, mice with CAC exhibited significantly reduced percentages of CD11b^+^Gr‐1^+^ MDSCs and GPNMB^+^ MDSCs almost to the level of NCs in all spleen, BM, and blood. Additionally, a similar pattern in GPNMB mRNA expression was detected in mouse BMCs (Figure 1C). These results indicate that CAC development in the mouse model is associated with expanded GPNMB‐expression MDSCs by a MyD88‐dependent pathway.

### 3.2 Suppressive effect of GPNMB‐expressing MDSCs on T cell proliferation and activation depends on the MyD88 pathway

**FIGURE 2:**
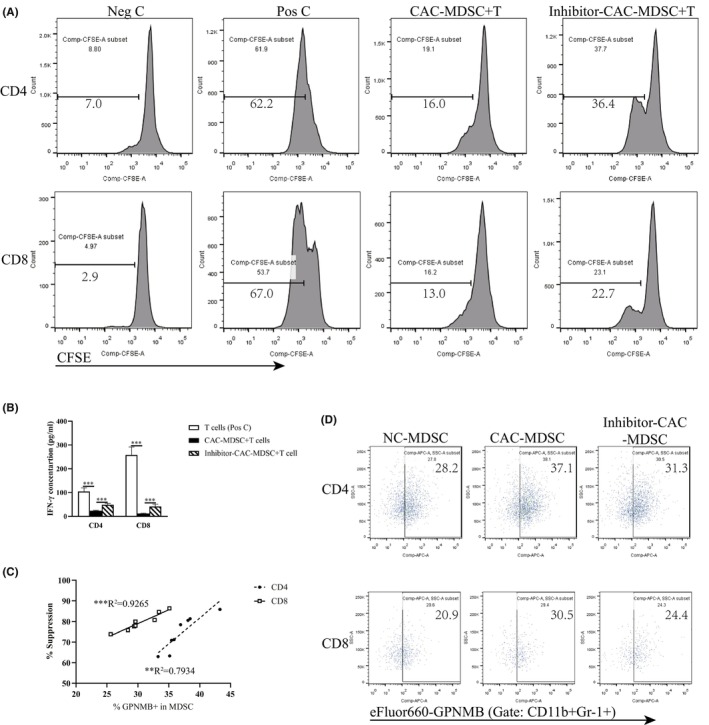
Suppressive effect of GPNMB^+^ MDSCs on T cell proliferation and IFN‐γ secretion depends on the MyD88 pathway. CD11b^+^Gr‐1^+^ MDSCs sorted from BM of mice were cocultured with CFSE‐labeled autologous CD4^+^ or CD8^+^ T cells in proportion (1:1) in the present of anti‐CD3/anti‐CD28‐antibody‐coated microbeads. (A) Percentage of CFSE‐labeled CD4^+^ T (upper panels) and CD8^+^ T (lower panels) cells after 72 h in culture, with no stimulation (negative control, Neg C), stimulation (positive control, Pos C) and cocultured MDSCs from mice with CAC (CAC‐MDSC) or MyD88 inhibitor‐treated mice with CAC (Inhibitor‐CAC‐MDSC). Data are expressed as the mean of each group. (B) Concentrations of IFN‐γ in the cultures were measured by ELISA. Data are expressed as the mean ± SD of each group. (C) Correlation of percentage of GPNMB^+^ cells among MDSCs from mice with CAC and their ability to suppress CD4^+^ or CD8^+^ T cell proliferation rates. Values (percentages) were plotted and analyzed for correlation coefficient *R* ^2^. (D) Population of GPNMB^+^ MDSCs (gated on CD11b^+^Gr‐1^+^ cells) sorted from the NC, CAC, Inhibitor‐CAC groups by flow cytometry cocultured with autologous CD4^+^ or CD8^+^ T cells. *N* = 8 per group. Data are expressed as the mean of each group. **p < 0.01; ***p < 0.001.

It is reported that GPNMB blockade restores the T‐cell response to antitumor by suppressing MDSC function.^17^ We here also detected the role of GPNMB pathway in the suppression effect of MDSCs on T cell function in the mice model of CAC. CD11b^+^Gr‐1^+^ MDSCs sorted from BMCs of mice with CAC were cocultured with autologous CD4^+^ or CD8^+^ T cells. CD4^+^ and CD8^+^ T cell proliferation rates were analyzed by CFSE assay in Figure 2A, which showed significant suppressive effects on CD4^+^ T cell proliferation rate [16.0 ± 5.3% vs. 62.2 ± 1.1% in positive control (PC), *p* = 0.0000] and CD8^+^ T cell proliferation rate (13.0 ± 4.5% vs. 67.0 ± 11.5% in PC, *p* = 0.0003). T cell activation was measured by IFN‐γ production in the cultures (Figure 2B), with significantly reduced concentration in the coculture groups (23.36 ± 2.64 pg/mL vs. 104.80 ± 15.32 pg/mL in PC of CD4^+^ T cells, *p* = 0.0000; 11.85 ± 2.00 pg/mL vs. 258.63 ± 33.05 pg/mL in PC of CD8^+^ T cells, *p* = 0.0000). The suppressor ability assessed by the percentages of suppression of CD4^+^ and CD8^+^ T cell proliferation rates. For CAC, GPNMB expression correlated positively with higher suppressor activity (*R* ^2^ = 0.7934/*p* = 0.0030 for CD4^+^ T cell proliferation and *R* ^2^ = 0.9265/*p* = 0.0001 for CD8^+^ T cell proliferation, Figure 2C).

However, the suppression of proliferation and activation of T cells were reduced by cocultured with MDSCs from the Inhibitor‐CAC mice (proliferation rate: 36.4 ± 8.3% of CD4^+^ T cells, *p* = 0.0000, and 22.7 ± 4.7% of CD8^+^ T cells, *p* = 0.0250, vs. CAC‐MDSC group, Figure 2A; IFN‐γ concentration: 48.84 ± 6.00 pg/mL of CD4^+^ T cells, *p* = 0.0002, and 41.64 ± 9.63 pg/mL of CD8^+^ T cells, *p* = 0.0009, Figure 2B), with decreased GPNMB‐expressing on MDSCs (31.3 ± 1.8% vs. 37.1 ± 3.0% on CAC‐MDSCs cocultured with CD4^+^ T cells, *p* = 0.0004; 24.4 ± 1.9% vs. 30.5 ± 3.1% on CAC‐MDSCs cocultured with CD8^+^T cells, *p* = 0.0003, Figure 2D). These results supported that GPNMB pathway mediated the suppression effect of MDSCs on T cell function in CAC and this process was dependent on the MyD88 pathway.

### 3.3 Suppressive effect of GPNMB‐expressing MDSCs on antitumor cytotoxicity of CD8 ^+^ T cells depends on the MyD88 pathway

**FIGURE 3:**
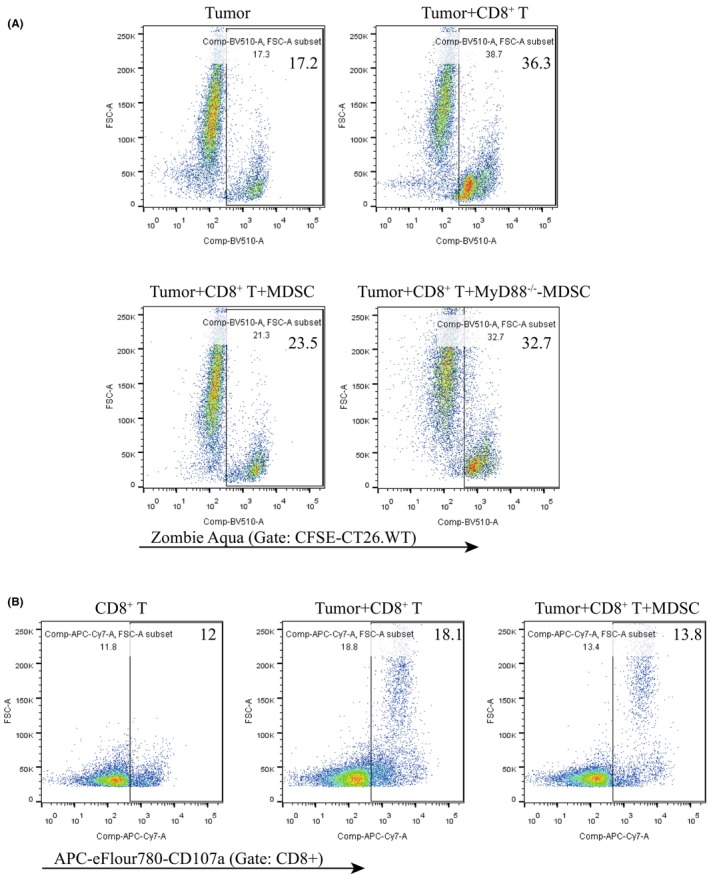
Suppressive effect of GPNMB^+^ MDSCs on antitumor cytotoxicity of CD8^+^ T cells depends on the MyD88 pathway. (A) Cytotoxicity of CT26.WT cells labeled by CFSE was detected by Zombie Aqua staining by flow cytometry cocultured with autologous CD8^+^ T cells w/o MDSCs. (B) Population of CD107a^+^ cells (gated on CD8^+^ T cells) in the cocultures by flow cytometry. *N* = 5 per group. Data are expressed as the mean of each group.

For CD8^+^ T cell cytotoxicity measurement, significantly increased 76.5% living CFSE‐CT26.WT cells remained after coculture with MDSC‐T cells, compared to those cocultured with CD8^+^ T cells (63.7% in CT26.WT cells, *p* = 0.0030) (Figure 3A). Additionally, the expression of CD107a on MDSC‐T cells were all significantly decreased after coculture with CT26.WT cells, compared with CD8^+^ T cells (13.8 ± 1.4% vs. 18.1 ± 1.3%, *p* = 0.0048, Figure 3B). To investigate the role of MyD88 signaling in MDSCs' suppression activity of CD8^+^ T cell antitumor cytotoxicity, MDSCs sorted from MyD88^−/−^ mice were cocultured with CD8^+^ T cells. The recovery killing effect of CD8^+^ T cells cocultured with MyD88^−/−^ MDSCs was observed with 67.3% living CFSE‐CT26.WT cells remained as compared to that of CD8^+^ T cells cocultured with wild type MDSCs (*p* = 0.0053, Figure 3A). Taken together, these findings indicated that MDSC suppresses CD8^+^ T cell antitumor cytotoxicity in CAC in a MyD88‐dependent manner.

### 3.4 MyD88 inhibitor suppressed MDSCs differentiation and GPNMB expression in vitro

**FIGURE 4:**
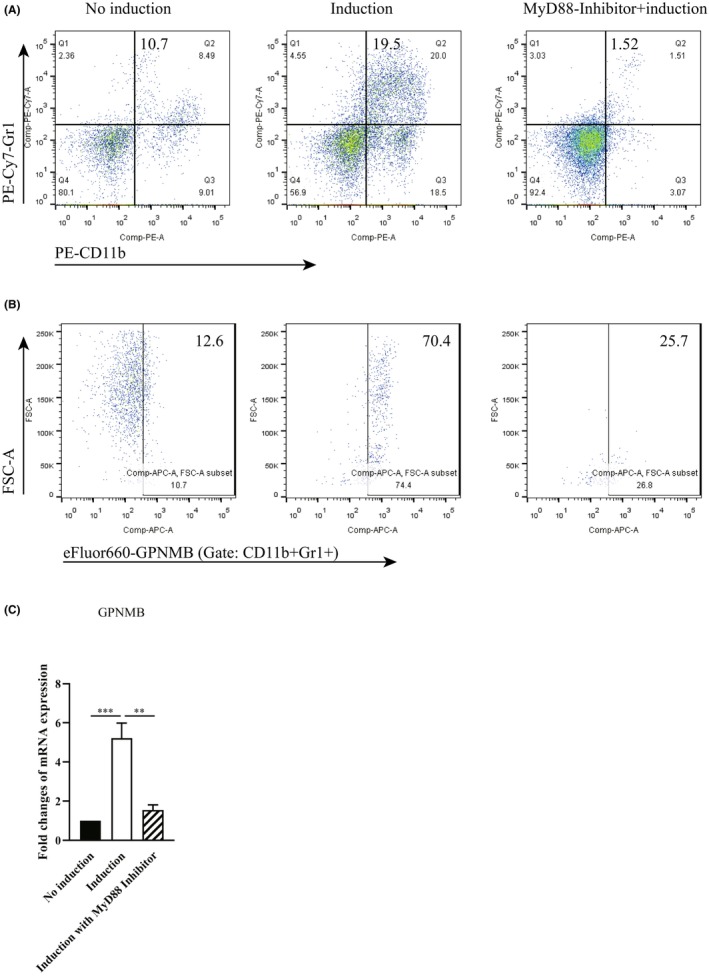
MyD88 Inhibitor suppressed MDSCs differentiation and GPNMB expression in vitro. (A) Population of CD11b^+^Gr‐1^+^ MDSCs induced from CD11b^−^ BMCs by GM‐CSF/LPS by flow cytometry. (B) Population of GPNMB^+^ cells (gated on CD11b^+^Gr‐1^+^ MDSCs) in the cultures by flow cytometry. (C) Relative levels of GPNMB mRNA transcripts in MDSCs induced in vitro examined by RT‐qPCR. Data of RT‐qPCR were normalized to β‐Actin expression in each sample, and the relative expression levels were compared with that in no induction group. Data are expressed as the mean ± SD of each group from three independent experiments. **p < 0.01; ***p < 0.001.

We have proved that blocking the MyD88 signaling pathway with the MyD88 inhibitor could significantly suppressed the differentiation of CD11b^−^ BMC induced to CD11b^+^Gr1^+^ MDSCs in vitro.^11^ Here, we further confirmed that the suppression was related to the expression of GPNMB on MDSCs. As shown in Figure 4B, the expression of GPNMB on MDSCs induced differentiation was significantly decreased after MyD88 inhibitor treatment (25.7%), compared with 70.4% in no‐inhibitor‐treated group, companied by a decreased in CD11b^+^Gr1^+^ MDSC population (1.52% vs. 19.5% in the no‐inhibitor‐treated group, Figure 4A). The mRNA expression of GPNMB in induced MDSCs was also significantly declined after the MyD88 inhibitor administration (Figure 4C). Thus, the GPNMB expression on MDSCs is associated with MyD88 signaling pathway activation.

## 4 DISCUSSION

TME is composed of malignant cells, various immune‐infiltrating cells (neutrophils, M_Φ_s, DCs, MDSCs, B and T lymphocytes, and natural killer cells), fibroblasts, endothelial cells, as well as the surrounding stroma.^19^ Interactions between TME and tumor cells could mostly influence tumor progression and metastasis.^20^ During tumorigenesis, there is a balance between protumor and antitumor immunity. In the process of tumor development, tumor cells evolve immunosuppressive mechanisms to escape immune surveillance, including expressing immunosuppressive molecules. One of these molecules is GPNMB whose role in enhancing immunosuppression has been increasingly confirmed in malignancy,^14^, ^21^ such as melanoma,^22^ breast cancer,^23^ glioma,^24^ and gastric cancer,^25^ which may offer an attractive target for cancer immunotherapy.^26^ An antibody anti‐GPNMB (glembatumumab vedotin)^27^ has been used in phase II clinical trials in the treatments of melanoma^28^, ^29^ and breast cancer.^30^

GPNMB is a highly glycosylated type 1 transmembrane protein that was first described in 1995 in melanoma cell lines.^31^ It is widely expressed in various tissues, such as the long bones, calvaria, bone marrow, skeletal muscle, skin, heart, lung, liver, pancreas, and placenta, and its expression is increased in cancer cells.^27^ It is encoded by the GPNMB gene located on chromosome 7p15^12^. GPNMB protein contains an extracellular domain, a single‐pass transmembrane domain, and a 53 amino acid cytoplasmic tail.^32^ It can be cleaved and release a soluble fragment that can bind to many partners and receptors, including integrins, heparin, Syndecan‐4, CD44, Na^+^‐K^+^‐ATPase, epidermal growth factor receptor, tyrosine kinase receptors, vascular endothelial growth factor receptor, and others,^21^, ^33^ and trigger multiple cellular responses.^34^

Human GPNMB^+^ MDSCs (CD14^+^HLA^−^DR^no/low^) and murine GPNMB^+^ MDSCs (CD11b^+^Gr1^+^) both play a considerable role in tumorigenesis, where they have been described as the significant suppressors of T cell function.^14^ GPNMB^+^CD11b^+^Gr1^+^ cells were massively expanded in melanoma‐bearing animals, and showed prominent suppressive effects on T cell activation both in vivo and in vitro. Administration of an anti‐GPNMB monoclonal Ab (mAb) or completely GPNMB knockout on CD11b^+^Gr1^+^ cells, could abrogate CD11b^+^Gr1^+^ cells expansion, impact their suppressor function on T cells, and diminish melanoma development in vivo.^35^ In clinical study, CD14^+^HLA^−^DR^no/low^ cells from metastatic melanoma patients showed increased GPNMB expression. These CD14^+^HLA^−^DR^no/low^ cells isolated from melanoma patients significantly reduced the IFN‐γ secretion of T cell in the cocultures of GPNMB^+^ MDSCs and autologous activated T cells.^36^ Similarly, GPNMB^+^ MDSCs isolated from CAC and pancreatic cancer patients exhibited significant suppressive effect on T cell function. However, administration of anti‐GPNMB mAb in the MDSC and T cell cocultures inhibited MDSC activity and restored the IFN‐γ production by T cells.^17^ Our results also dedicated that GPNMB‐expressing MDSCs are widely expanded in the mouse model of CAC (Figure 1), and those from mice of CAC have significant suppressive effects on T cells proliferation and IFN‐γ production (Figure 2). Additionally, our findings expressed the suppressive effect of MDSC on antitumor cytotoxicity of CD8^+^ T cell (Figure 3) for the first time, and illustrated the positive correlation between the suppressor activity of MDSC and GPNMB expression level on it. Thus, here we provide valuable information that GPNMB regulation on MDSC may serve as a tumor‐targeting therapy for CAC.

There is limited research on the regulatory and effect molecular mechanisms of GPNMB expressing on MDSCs. It is reported that microphthalmia transcription factor (MITF) binds and trans‐activates the *Gpnmb* promoter, and drives GPNMB expression.^37^ Through enhancing PI3K/AKT/mTOR pathway signaling and β‐catenin activity, GPNMB promotes breast tumor initiation and growth mediated by Wnt‐1 signaling.^38^ GPNMB mechanistically augments IL‐33‐mediated tumorigenesis by activating CD44 receptor.^39^ Binding of MDSC‐derived GPNMB to SD‐4 on activated T cells greatly blocked CD4^+^ and CD8^+^ T cell responses.^13^ TLR activation by agonists induces amplified GPNMB/SD‐4 pathway, which is a direct result of NF‐κB binding to the promoter and induction of de novo transcription.^40^ In our study, blockage of TLR/MyD88 signaling pathway results in less of GPNMB‐expressing MDSCs expanding in CAC (Figure 1), less of suppression effect of GPNMB‐expressing MDSCs on T cell proliferation, activation (Figure 2), and antitumor cytotoxicity (Figure 3), and less of MDSCs differentiation and GPNMB expression in vitro (Figure 4). Our results firstly proved the relationship between TLR/MyD88 signaling pathway activation in innate immunity and MDSC's suppressive function on antitumor T‐cell adoptive immunity. Therefore, our studies provide strong support for the suppressive effect of MDSC on T cell antitumor immunity in CAC via GPNMB in a MyD88‐dependent manner.

## 5 CONCLUSIONS

In summary, we have uncovered that the CAC's immune silence derive from GPNMB‐expressing MDSC‐mediated T cell suppression, and the suppressive effect of MDSCs is positively correlated with GPNMB expression level. TLR/MyD88 signaling pathway is proved to play an essential role in MDSC‐mediated immune escape in CAC development and is a promising focus for revealing the mechanisms of MDSC that facilitate immunosuppression and tumor progression.

## AUTHOR CONTRIBUTIONS

**Bo Wang:** Conceptualization (equal); funding acquisition (equal). **Lu Wang:** Data curation (equal); formal analysis (equal); investigation (equal); methodology (equal). **Runshi Shang:** Investigation (equal); methodology (equal). **Lin Xie:** Conceptualization (equal); data curation (equal); funding acquisition (equal); investigation (equal); methodology (equal); supervision (equal); writing – original draft (equal); writing – review and editing (equal).

## FUNDING INFORMATION

This work was supported by the National Natural Science Foundations of China (Grant #82273309); the Hubei Provincial Natural Science Foundation of China (2021CFB401); the Hubei Province health and family planning scientific research project (WJ2023M014); and Open Project of the Key Laboratory of Organ Transplantation, Ministry of Education, and NHC (2020QYKF02).

## CONFLICT OF INTEREST STATEMENT

The authors declare no conflict of interest.

## ETHICS STATEMENT

This study was carried out in accordance with the recommendations and guidelines issued by the Ethics Committee of Huazhong University of Science and Technology (Wuhan, China). Written informed consent for publication of this paper was obtained from the Tongji Hospital, Huazhong University of Science and Technology (No. TJH‐201803001) and all authors.

## Data Availability

The data that support the findings of this study are available on request from the corresponding author, [lx], on reasonable request.

## References

[1] Wang Y , Ding Y , Deng Y , Zheng Y , Wang S . Role of myeloid‐derived suppressor cells in the promotion and immunotherapy of colitis‐associated cancer. J Immunother Cancer. 2020;8(2):e000609. doi:10.1136/jitc-2020-000609 33051339 PMC7555106

[2] Safarzadeh E , Orangi M , Mohammadi H , Babaie F , Baradaran B . Myeloid‐derived suppressor cells: important contributors to tumor progression and metastasis. J Cell Physiol. 2018;233(4):3024‐3036. doi:10.1002/jcp.26075 28661031

[3] Li K , Shi H , Zhang B , et al. Myeloid‐derived suppressor cells as immunosuppressive regulators and therapeutic targets in cancer. Signal Transduct Target Ther. 2021;6(1):362. doi:10.1038/s41392-021-00670-9 34620838 PMC8497485

[4] Haile LA , von Wasielewski R , Gamrekelashvili J , et al. Myeloid‐derived suppressor cells in inflammatory bowel disease: a new immunoregulatory pathway. Gastroenterology. 2008;135(3):871‐881. doi:10.1053/j.gastro.2008.06.032 18674538

[5] Guven Maiorov E , Keskin O , Gursoy A , Nussinov R . The structural network of inflammation and cancer: merits and challenges. Semin Cancer Biol. 2013;23(4):243‐251. doi:10.1016/j.semcancer.2013.05.003 23712403

[6] Skabytska Y , Wolbing F , Gunther C , et al. Cutaneous innate immune sensing of toll‐like receptor 2‐6 ligands suppresses T cell immunity by inducing myeloid‐derived suppressor cells. Immunity. 2014;41(5):762‐775. doi:10.1016/j.immuni.2014.10.009 25456159

[7] Delano MJ , Scumpia PO , Weinstein JS , et al. MyD88‐dependent expansion of an immature GR‐1(+)CD11b(+) population induces T cell suppression and Th2 polarization in sepsis. J Exp Med. 2007;204(6):1463‐1474. doi:10.1084/jem.20062602 17548519 PMC2118626

[8] Arora M , Poe SL , Oriss TB , et al. TLR4/MyD88‐induced CD11b+gr‐1 int F4/80+ non‐migratory myeloid cells suppress Th2 effector function in the lung. Mucosal Immunol. 2010;3(6):578‐593. doi:10.1038/mi.2010.41 20664577 PMC2958091

[9] Liu Y , Xiang X , Zhuang X , et al. Contribution of MyD88 to the tumor exosome‐mediated induction of myeloid derived suppressor cells. Am J Pathol. 2010;176(5):2490‐2499. doi:10.2353/ajpath.2010.090777 20348242 PMC2861113

[10] Hong EH , Chang SY , Lee BR , et al. Blockade of Myd88 signaling induces antitumor effects by skewing the immunosuppressive function of myeloid‐derived suppressor cells. Int J Cancer. 2013;132(12):2839‐2848. doi:10.1002/ijc.27974 23184679

[11] Wang L , Hu D , Xie B , Xie L . Blockade of Myd88 signaling by a novel MyD88 inhibitor prevents colitis‐associated colorectal cancer development by impairing myeloid‐derived suppressor cells. Invest New Drugs. 2022;40(3):506‐518. doi:10.1007/s10637-022-01218-6 35089465 PMC9098617

[12] van der Lienden MJC , Gaspar P , Boot R , Aerts J , van Eijk M . Glycoprotein non‐metastatic protein B: an emerging biomarker for lysosomal dysfunction in macrophages. Int J Mol Sci. 2018;20(1):66. doi:10.3390/ijms20010066 30586924 PMC6337583

[13] Chung JS , Tamura K , Akiyoshi H , Cruz PD Jr , Ariizumi K . The DC‐HIL/syndecan‐4 pathway regulates autoimmune responses through myeloid‐derived suppressor cells. J Immunol. 2014;192(6):2576‐2584. doi:10.4049/jimmunol.1301857 24516197 PMC3957202

[14] Lazaratos AM , Annis MG , Siegel PM . GPNMB: a potent inducer of immunosuppression in cancer. Oncogene. 2022;41(41):4573‐4590. doi:10.1038/s41388-022-02443-2 36050467

[15] Taya M , Hammes SR . Glycoprotein non‐metastatic melanoma protein B (GPNMB) and cancer: a novel potential therapeutic target. Steroids. 2018;133:102‐107. doi:10.1016/j.steroids.2017.10.013 29097143 PMC6166407

[16] Colombo MP . Is GPNMB the Achilles' heel of Mo‐MDSC while marking their suppressive activity? Clin Cancer Res. 2019;25(2):453‐454. doi:10.1158/1078-0432.CCR-18-2334 30166442

[17] Kobayashi M , Chung JS , Beg M , et al. Blocking Monocytic myeloid‐derived suppressor cell function via anti‐DC‐HIL/GPNMB antibody restores the in vitro integrity of T cells from cancer patients. Clin Cancer Res. 2019;25(2):828‐838. doi:10.1158/1078-0432.CCR-18-0330 30049749 PMC7315386

[18] Xie L , Jiang FC , Zhang LM , et al. Targeting of MyD88 Homodimerization by novel synthetic inhibitor TJ‐M2010‐5 in preventing colitis‐associated colorectal cancer. J Natl Cancer Inst. 2016;108(4):djv364. doi:10.1093/jnci/djv364 26712311

[19] Quail DF , Joyce JA . Microenvironmental regulation of tumor progression and metastasis. Nat Med. 2013;19(11):1423‐1437. doi:10.1038/nm.3394 24202395 PMC3954707

[20] Ganesh K , Massague J . Targeting metastatic cancer. Nat Med. 2021;27(1):34‐44. doi:10.1038/s41591-020-01195-4 33442008 PMC7895475

[21] Saade M , Araujo de Souza G , Scavone C , Kinoshita PF . The role of GPNMB in inflammation. Front Immunol. 2021;12:674739. doi:10.3389/fimmu.2021.674739 34054862 PMC8149902

[22] Tomihari M , Chung JS , Akiyoshi H , Cruz PD Jr , Ariizumi K . DC‐HIL/glycoprotein Nmb promotes growth of melanoma in mice by inhibiting the activation of tumor‐reactive T cells. Cancer Res. 2010;70(14):5778‐5787. doi:10.1158/0008-5472.CAN-09-2538 20570888 PMC2905472

[23] Rose AA , Siegel PM . Osteoactivin/HGFIN: is it a tumor suppressor or mediator of metastasis in breast cancer? Breast Cancer Res. 2007;9(6):403. doi:10.1186/bcr1791 18086324 PMC2246174

[24] Bao G , Wang N , Li R , Xu G , Liu P , He B . Glycoprotein non‐metastaticmelanoma protein B promotes glioma motility and angiogenesis through the Wnt/beta‐catenin signaling pathway. Exp Biol Med (Maywood). 2016;241(17):1968‐1976. doi:10.1177/1535370216654224 27334625 PMC5068464

[25] Ren F , Zhao Q , Liu B , et al. Transcriptome analysis reveals GPNMB as a potential therapeutic target for gastric cancer. J Cell Physiol. 2020;235(3):2738‐2752. doi:10.1002/jcp.29177 31498430

[26] Zhou LT , Liu FY , Li Y , Peng YM , Liu YH , Li J . Gpnmb/osteoactivin, an attractive target in cancer immunotherapy. Neoplasma. 2012;59(1):1‐5. doi:10.4149/neo_2012_001 22017590

[27] Rose AAN , Biondini M , Curiel R , Siegel PM . Targeting GPNMB with glembatumumab vedotin: current developments and future opportunities for the treatment of cancer. Pharmacol Ther. 2017;179:127‐141. doi:10.1016/j.pharmthera.2017.05.010 28546082

[28] Hasanov M , Rioth MJ , Kendra K , et al. A phase II study of Glembatumumab Vedotin for metastatic uveal melanoma. Cancers (Basel). 2020;12(8):2270. doi:10.3390/cancers12082270 32823698 PMC7465139

[29] Ott PA , Pavlick AC , Johnson DB , et al. A phase 2 study of glembatumumab vedotin, an antibody‐drug conjugate targeting glycoprotein NMB, in patients with advanced melanoma. Cancer. 2019;125(7):1113‐1123. doi:10.1002/cncr.31892 30690710

[30] Vahdat LT , Schmid P , Forero‐Torres A , et al. Glembatumumab vedotin for patients with metastatic, gpNMB overexpressing, triple‐negative breast cancer ("METRIC"): a randomized multicenter study. NPJ Breast Cancer. 2021;7(1):57. doi:10.1038/s41523-021-00244-6 34016993 PMC8137923

[31] Weterman MA , Ajubi N , van Dinter IM , et al. Nmb, a novel gene, is expressed in low‐metastatic human melanoma cell lines and xenografts. Int J Cancer. 1995;60(1):73‐81. doi:10.1002/ijc.2910600111 7814155

[32] Hoashi T , Sato S , Yamaguchi Y , Passeron T , Tamaki K , Hearing VJ . Glycoprotein nonmetastatic melanoma protein b, a melanocytic cell marker, is a melanosome‐specific and proteolytically released protein. FASEB J. 2010;24(5):1616‐1629. doi:10.1096/fj.09-151019 20056711 PMC2879953

[33] Tsou PS , Sawalha AH . Glycoprotein nonmetastatic melanoma protein B: a key mediator and an emerging therapeutic target in autoimmune diseases. FASEB J. 2020;34(7):8810‐8823. doi:10.1096/fj.202000651 32445534 PMC7501235

[34] Rose AA , Annis MG , Dong Z , et al. ADAM10 releases a soluble form of the GPNMB/Osteoactivin extracellular domain with angiogenic properties. PLoS One. 2010;5(8):e12093. doi:10.1371/journal.pone.0012093 20711474 PMC2919417

[35] Chung JS , Tamura K , Cruz PD Jr , Ariizumi K . DC‐HIL‐expressing myelomonocytic cells are critical promoters of melanoma growth. J Invest Dermatol. 2014;134(11):2784‐2794. doi:10.1038/jid.2014.254 24936834 PMC4199867

[36] Turrentine J , Chung JS , Nezafati K , et al. DC‐HIL+ CD14+ HLA‐DR no/low cells are a potential blood marker and therapeutic target for melanoma. J Invest Dermatol. 2014;134(11):2839‐2842. doi:10.1038/jid.2014.248 24933321 PMC4199894

[37] Loftus SK , Antonellis A , Matera I , et al. Gpnmb is a melanoblast‐expressed, MITF‐dependent gene. Pigment Cell Melanoma Res. 2009;22(1):99‐110. doi:10.1111/j.1755-148X.2008.00518.x 18983539 PMC2714741

[38] Maric G , Annis MG , MacDonald PA , et al. GPNMB augments Wnt‐1 mediated breast tumor initiation and growth by enhancing PI3K/AKT/mTOR pathway signaling and beta‐catenin activity. Oncogene. 2019;38(26):5294‐5307. doi:10.1038/s41388-019-0793-7 30914799

[39] Liguori M , Digifico E , Vacchini A , et al. The soluble glycoprotein NMB (GPNMB) produced by macrophages induces cancer stemness and metastasis via CD44 and IL‐33. Cell Mol Immunol. 2021;18(3):711‐722. doi:10.1038/s41423-020-0501-0 32728200 PMC8027814

[40] Smith MF Jr , Novotny J , Carl VS , Comeau LD . Helicobacter pylori and toll‐like receptor agonists induce syndecan‐4 expression in an NF‐kappaB‐dependent manner. Glycobiology. 2006;16(3):221‐229. doi:10.1093/glycob/cwj061 16319082 PMC1370916

